# Peripheral T cell immune repertoire is associated with the outcomes of acute spontaneous intracerebral hemorrhage

**DOI:** 10.3389/fneur.2024.1371830

**Published:** 2024-03-14

**Authors:** Rui Zhang, Li Wang, Jiapo Zhang, Xiufang Zhang, Peng Wang

**Affiliations:** ^1^Department of Anesthesiology, The Affiliated Hospital of Qingdao University, Qingdao, Shandong, China; ^2^Department of Emergency Medicine, Xiang’an Hospital of Xiamen University, School of Medicine, Xiamen University, Xiamen, Fujian, China

**Keywords:** intracerebral hemorrhage, T cell, T cell receptor, IR-seq, immune repertoire

## Abstract

Systematic immune responses have been identified in patients with acute spontaneous intracerebral hemorrhage (ICH). T cells have been established to participate in central nervous system damage and repair following brain injury. However, their contribution to the prognosis of patients with ICH remains to be elucidated. In this study, peripheral blood mononuclear cells (PBMCs) were collected from 45 patients with acute spontaneous ICH (<24 h from symptom onset). Our results exposed significant negative correlations between hematoma volume/white blood cell (WBC) density and Glasgow Coma Scale (GCS) score. Contrastingly, lymphocyte density was negatively correlated with hematoma volume and positively correlated with GCS score. Moreover, flow cytometry determined that ICH activated T cells despite their proportion being lower in blood. Afterward, immune repertoire sequencing (IR-seq) revealed a significant decrease in VJ, VDJ usage, and TCR clonotypes in ICH patients. Finally, variations in the complementarity-determining region 3 (CDR3) amino acid (aa) were also detected in ICH patients. This study reveals the occurrence of peripheral T-cell diminishment and activation in response to acute hematoma. ICH lesion also alters the T cell receptor (TCR) immune repertoire, which is associated with patient prognosis.

## Introduction

Acute spontaneous intracerebral hemorrhage (ICH) is a prevalent type of stroke (10%–15% of all strokes) that afflicts approximately 2 million people worldwide annually (1). It elevates intracranial pressure and induces neurologic deficits, leading to high morbidity and mortality due to limited therapeutic approaches, and only one-fifth of survivors regain their independence after 6 months (2). Accumulating evidence suggests that surgical removal of the blood clot does not benefit patients (2, 3). Consequently, there is an urgent need to discover novel approaches in order to enhance functional recovery.

Despite being “immune privileged,” T cells are implicated in cognitive and social brain function under both physiological and pathological conditions (4). Hematoma formation disrupts the blood–brain barrier (BBB), resulting in inflammatory responses and lymphocyte extravasation from peripheral blood into the central nervous system (CNS), eventually eliciting brain injury (5). Compelling evidence indicates that the activity of infiltrating T cells unequivocally contributes to the progression of neuroinflammation following CNS injury (6). However, the potential mechanisms underlying the infiltration and role of T cells in ICH are currently underexplored. Recent studies present two conflicting hypotheses (beneficial or harmful) regarding the effect of T cells after CNS injury, potentially attributed to the type of infiltrating T cells and the stage of the disease (5, 7, 8).

As is well documented, the levels of various T cell subsets (including regulatory T cells and helper T 17 cells) and cytokines (including IL-6, IL-17, IL-23, TNF-α, IL-4, IL-10, and TGF-β) are higher in the peripheral blood of ICH patients (9). However, the crosstalk between T cells, cytokines, and clinical outcomes in ICH patients remains elusive. The origin and type of T cells can be identified via T cell receptor (TCR) immune repertoire sequencing (IR-seq) using next-generation sequencing (NSG) (10). TCR is composed of variable (V), diversity (D), joining (J), and constant (C) domains, wherein the area from the terminus of the V domain to the beginning of the J domain is referred to as the complementarity-determining region 3 (CDR3) (11). CDR3 is a critical area that recognizes and binds specific antigens, thereby determining TCR specificity and clonotype (12, 13). Our previous studies inferred that T cells can sense external stimuli and preserve endogenous homeostasis, leading to the reconstitution of the TCR repertoire under disease status (14–16). Nevertheless, fluctuations in the TCR repertoire and its association with outcomes in ICH have not been elucidated so far.

According to the findings of previous studies, we hypothesized an essential evolution of the TCR repertoire in response to ICH-induced immune abnormalities. In the present study, the correlation between the total number of different peripheral blood cells and hematoma volume/Glasgow Coma Scale (GCS) score was initially assessed. Briefly, peripheral blood mononuclear cells (PBMCs) from patients with ICH and healthy controls were collected to measure the T cell state via flow cytometry. Then, NGS was utilized to monitor the profile of the TCR repertoire in ICH-associated PBMCs within 24 h from symptom onset. Our work identified a shift in the TCR repertoire in ICH, which is closely associated with patient prognosis.

## Materials and methods

### Subjects

This study belongs to basic experimental research. A total of 45 patients (26 male and 19 female; mean age = 59.9 ± 12.4 years) diagnosed with acute spontaneous ICH (<24 h from symptom onset) were admitted to the Department of Emergency Medicine, Xiang’an Hospital of Xiamen University from May 2021 to May 2022. All patients underwent head CT scan (Supplementary Figure S1) and GCS evaluation within 6 h of hospital admission. Exclusion criteria were (a) non-parenchymal hemorrhages (isolated subarachnoid hemorrhage, epidural or subdural hematoma), (b) ICH attributed to a clearly defined cause (trauma, structural vascular lesions, neoplasms, vasculitis, infection), (c) unavailable head CT scan within 6 h, (d) prior administration of drugs or surgical intervention before hospital admission, (e) infectious, autoimmune and chronic disease. The volume of the hematoma was calculated as previously described using CT images by two experienced neurologists (17). A major proportion (>90%) of patients belong to hypertension with basal ganglia hematoma. For biological experiments, the samples were randomly selected from these patients. Additionally, 10 healthy individuals (5 males and 5 females; mean age = 60.2 ± 7.8 years) without clinical signs of ICH were included as controls. The study protocol was approved by the Institutional Ethical Commission for the School of Medicine at Xiamen University. Written informed consent was obtained from all participants in accordance with the principles of the Declaration of Helsinki.

### PBMCs collection and flow cytometry

Human blood samples were collected in EDTA-treated anticoagulant tubes from ICH patients within 24 h after ICH onset. For PBMC isolation, blood and Ficoll (Solarbio Life Sciences, Beijing, China) were mixed (volume ratio 1:1) and centrifuged at 1,000 g for 30 min. Then, the isolated cells were washed 3 times, followed by staining with fluorescently conjugated antibodies against FITC-CD3 (clone: OKT3) and PE-CD38 (clone: HIT2) from BioLegend. Lastly, the stained cells were examined using a BD Aria III machine and analyzed with FlowJo software 10.6.2 version (TreeStar).

### Immune repertoire sequencing and data analysis

Total RNA was isolated from PBMCs using a SPARKeasy RNA Extraction Kit (Sparkiade Biotechnology Co., Ltd., Shandong, China) according to the manufacturer’s specifications. A Transcriptor First Strand cDNA Synthesis Kit (LABLEAD, Beijing, China) was employed to reverse-transcribe RNA to cDNA using a T1000 Thermal Cycler (Bio-Rad Inc., Hercules, CA). For TCR immune repertoire library construction, a two-round nested amplicon PCR was performed using specific primers as previously described. Purified amplicons were paired-end sequenced (PE150) on the Illumina HiSeq X Ten platform (Illumina, San Diego, CA).

For immune repertoire data analysis, Blast Plus was utilized to identify TCR β chain V, D, and J genes in each sequence based on the TCR reference genome sourced from the International Immunogenetics Information System (IMGT)/GeneDB database. VDJmatch 1.2.2, VDJtools 1.2.1 package and VDJdb were used to identify the usage and clonotype of V, D, and J genes. The motif of CDR3 aa was identified using the ggseqlogo 0.1 package.

### Statistical analysis

Statistical analyses were conducted using Prism 8.0 software (GraphPad Software), and data were presented as means ± standard deviations (SD). For immune repertoire data, a normality check has been done using Shapiro–Wilk test. The results showed that immune repertoire data (frequency, clonotypes, Chao1 and Gini coefficient) followed normal distribution. The two-tailed unpaired Student’s *t*-test was used for the comparison of two independent groups. Correlation analysis was performed using Spearman’s rank correlation test. *p* values less than 0.05 were considered statistically significant.

## Results

### Acute spontaneous ICH reduces T-cell abundance in PBMCs

**Figure 1:**
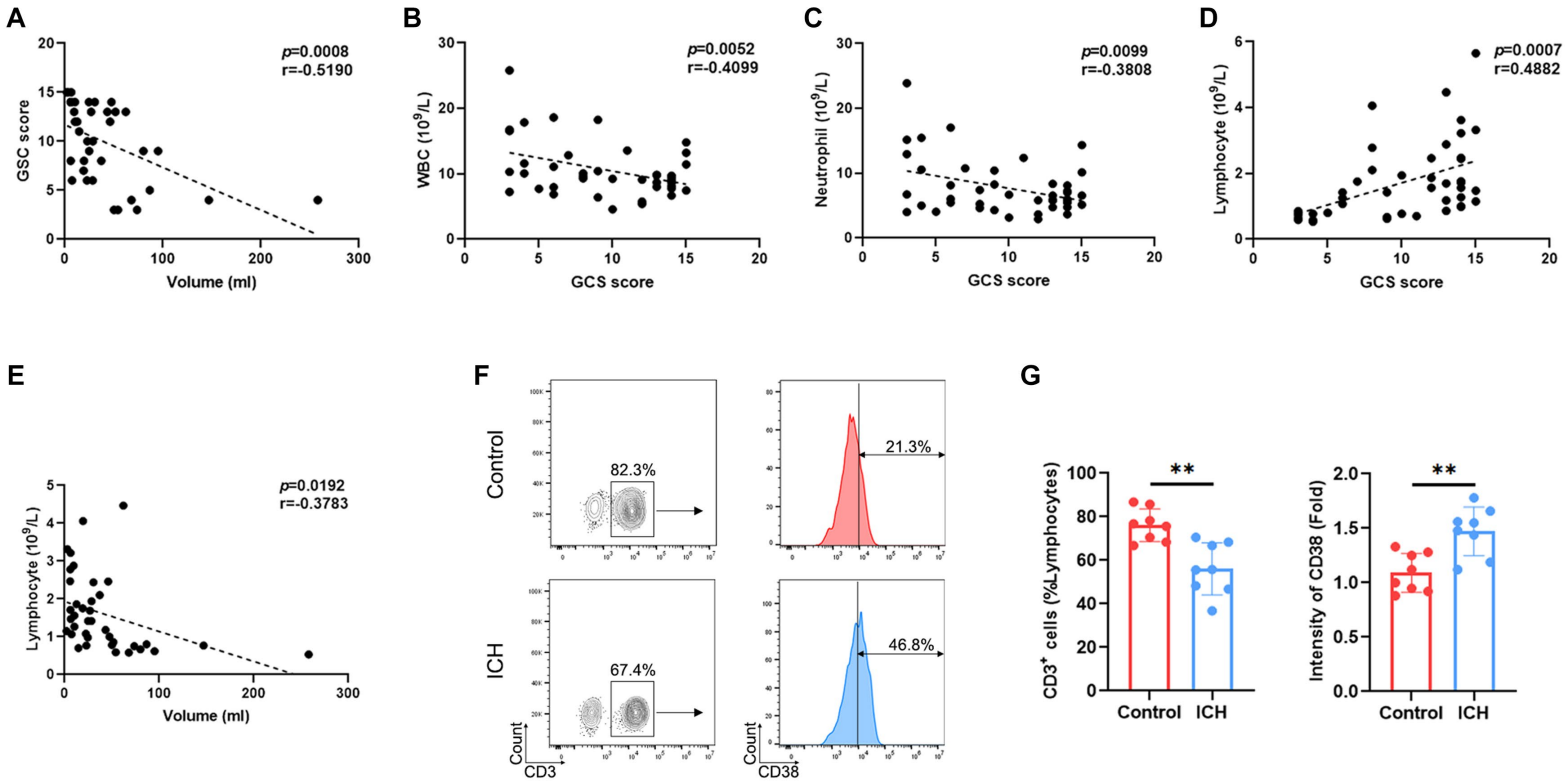
ICH leads to abnormal peripheral T-cell responses. **(A)** Correlation analysis between GCS score and hematoma volume. *n* = 38. **(B–D)** Correlation analysis between the count of WBCs/neutrophils/lymphocytes and GCS score. *n* = 45. **(E)** Correlation analysis between the lymphocyte count and hematoma volume. *n* = 45. **(F)** Representative flow cytometry image of CD3-positive cells and the expression levels of CD3-gated CD38. *n* = 8. **(G)** The percentage and CD38 fluorescence intensities of T cells. Data represent the results of three to five independent experiments. ^**^p < 0.01.

Emerging evidence supports the notion that the hematoma volume reflects the severity and the prognosis of ICH. Indeed, our results demonstrated a negative correlation between GCS score and hematoma volume (Figure 1A). Interestingly, the GCS score was negatively correlated with the total count of WBC/neutrophils (Figures 1B,C) and positively correlated with lymphocyte counts (Figure 1D) in ICH-associated PBMCs. Similarly, a negative correlation was identified between hematoma volume and the lymphocyte count (Figure 1E). However, the total number of other cell subsets was not associated with GCS score or hematoma volume (Supplementary Figure S2). Moreover, flow cytometry determined a substantial decrease in the proportion of T cells and an increase in that of activated T cells (Figures 1F,G).

### The patterns of V, D, and J genes in ICH

**Figure 2:**
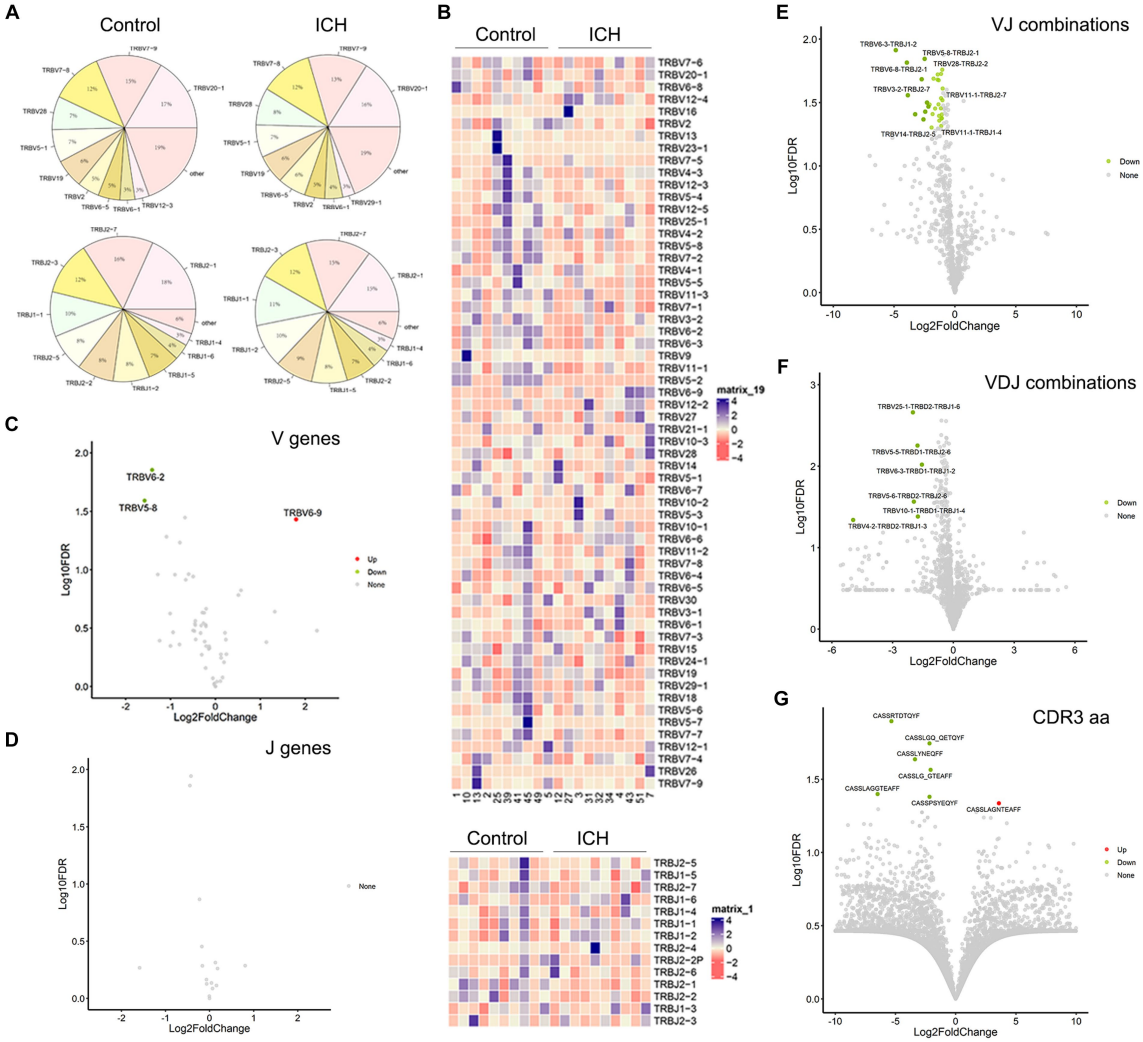
The usage of V gene, J gene and CDR3 aa. **(A)** The distribution of the top 10 V and J genes. *n* = 10. **(B)** Heatmaps of hierarchical clustering of V and J gene. *n* = 10. **(C,D)** Volcano plots of frequency of the V and J genes. *n* = 10. **(E–G)** Volcano plots of frequency of VJ combinations, VDJ combinations, and CDR3 aa. *n* = 10. Data represent the results of three to five independent experiments.

PBMC IR-seq from 10 control and 10 ICH patients were used to detect the TCR repertoire, yielding 4.35 × 10^6^~7.93 × 10^6^ productively TCR β chain blast reads per sample, with matching rates within the range of 65.03%~90.34% (Supplementary Table S1). A total of 52~58 V genes and 13~14 J genes were identified across all samples. Generally, the type and top usages of V/J were similar between the two groups (Figures 2A,B). On the other hand, significant differences were noted in the frequency of TRBV6-2, TRBV5-8, and TRBV6-9 (Figures 2C,D). Besides, the composition of paired VJ and VDJ usages was also analyzed. A total of 535~618 VJ usages and 821~1,107 VDJ usages were detected across all samples. Importantly, ICH significantly limited the frequency of several VJ and VDJ usages (Figures 2E,F). While a total of 32,123~76,735 CDR3 aa were identified, few CDR3 aa frequencies were significantly different between the control and ICH groups (Figure 2G).

### ICH decreases the diversity of the T cell immune repertoire

**Figure 3:**
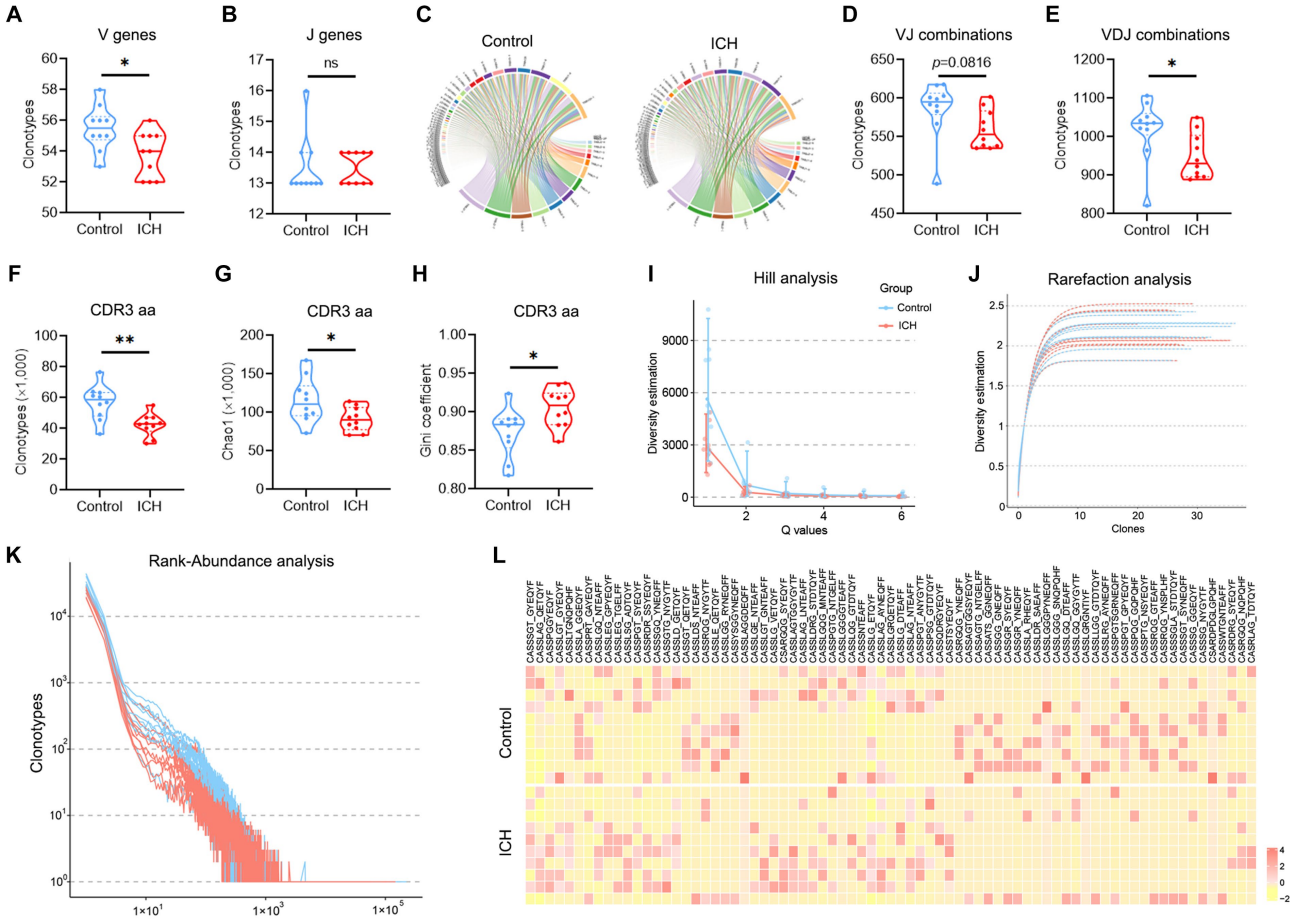
A significantly decreased TCR diversity was identified in ICH. **(A,B)** Clonotype of the V and J genes. *n* = 10. **(C)** Representative circle plot of paired VJ combinations. **(D,E)** Clonotype of VJ and VDJ combinations. *n* = 10. **(F–H)** Clonotype, Chao1, and Gini coefficient of CDR3 aa. *n* = 10. **(I–K)** Hill analysis, Rarefaction analysis, and Rank-Abundance analysis of CDR3 aa. *n* = 10. **(L)** Heatmap of the hierarchical clustering of similar CDR3 aa clusters. *n* = 10. ^*^p < 0.05, ^**^p < 0.01. Data represent the results of three to five independent experiments.

The types of CDR3aa clonotype represent the diversity of the TCR immune repertoire. Of note, ICH markedly decreased the clonotype of V, J, VJ, VDJ, and CDR3aa despite their usage frequencies being comparable (Figures 3A–E). Subsequently, multiple parameters were used to evaluate the richness and diversity of the TCR immune repertoire. Chao1 index and Gini coefficient indicated a lower CDR3 aa abundance in ICH-associated PBMCs (Figures 3F–H). Likewise, Hill analysis, Rarefaction analysis, and Rank-Abundance analysis also identified a lower diversity of TCR clonotypes in PBMCs from ICH patients (Figures 3I–K). Furthermore, similar CDR3aa were clustered to compare their expression between the two groups. As anticipated, there was a significantly sparse distribution in the ICH group (Figure 3L).

### TCR diversity is associated with the outcome of ICH

**Figure 4:**
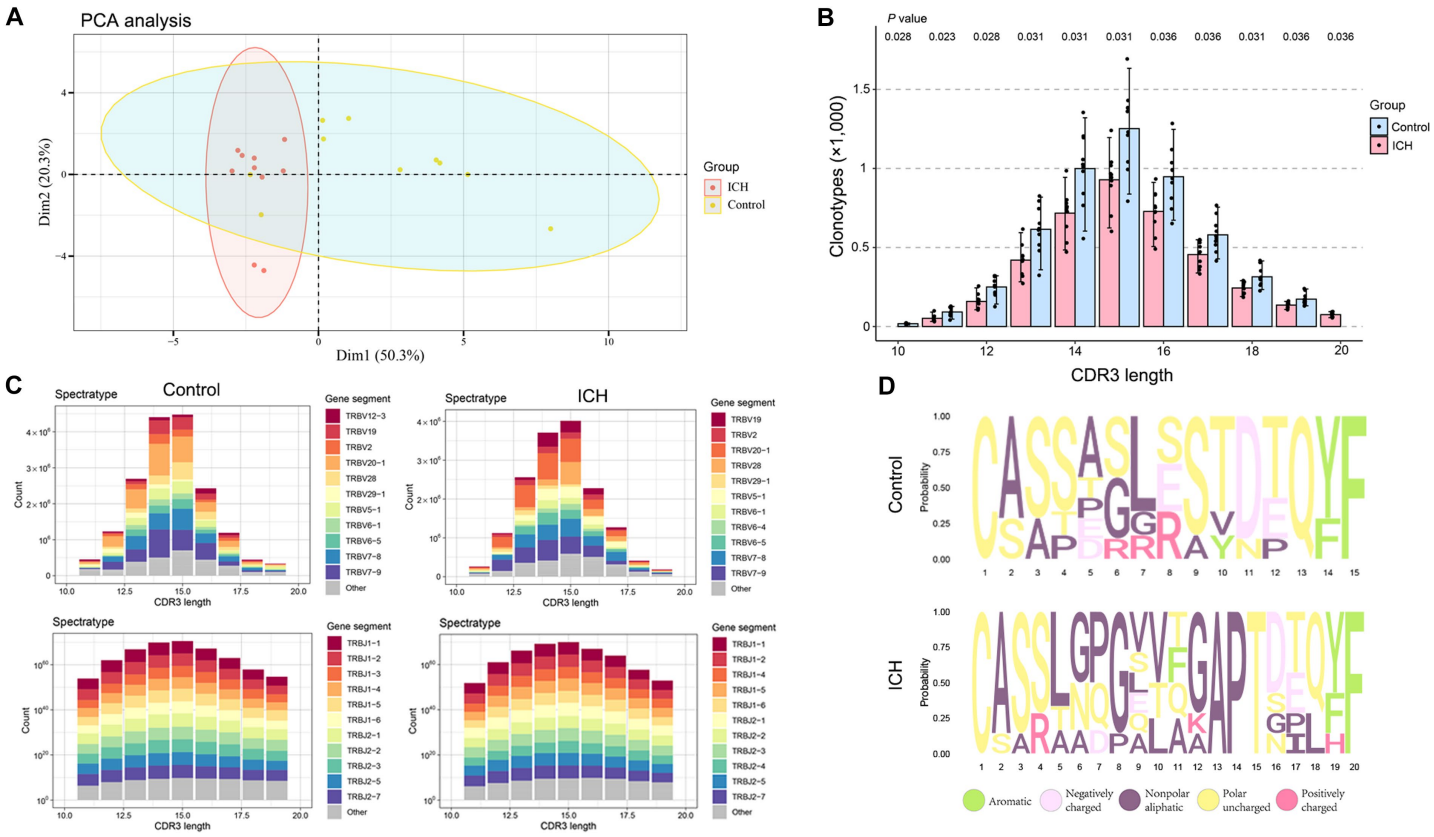
ICH results in CDR3 aa reconstitution. **(A)** PCA analysis of CDR3 aa. *n* = 10. **(B)** Comparison of clonotypes of different CDR3 aa lengths. *n* = 10. **(C)** The spectratype of the V and J genes in different CDR3 aa lengths. *n* = 10. **(D)** High-frequency (top 50%) CDR3 aa motifs. Data represent the results of three to five independent experiments.

**Figure 5:**
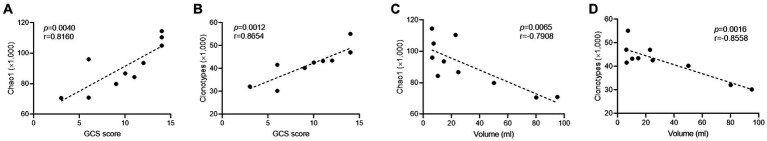
TCR diversity predicts ICH severity. **(A,B)** Correlation analysis between Chao1, clonotype of CDR3 aa, and GCS score. *n* = 10. **(C,D)** Correlation analysis between Chao1, clonotype of CDR3 aa, and hematoma volume. *n* = 10. Data represent the results of three to five independent experiments.

Principal component analysis revealed that ICH resulted in a significantly different CDR3 aa profile characterized by a centralized distribution, signaling aberrant composition of CDR3 aa clonotypes (Figure 4A). Noteworthily, a substantial increase in CDR3 aa length was found after ICH, although the usages of V and J genes were similar across different CDR3 aa lengths (Figures 4B,C). Furthermore, the specific motif in high-frequency CDR3 aa (top 50%) was examined. Of note, a significantly different CDR3 motif was identified in the ICH group (Figure 4D). However, the proportion of high-frequency CDR3 aa was comparable between the two groups (Supplementary Figure S3). To investigate the correlation between the profile of CDR3 aa and outcomes of acute spontaneous ICH, correlation analyses of clonotype or chao1 of CDR3 aa and GCS score or hematoma volume were executed. On the one hand, a significant positive correlation was observed between CDR3 aa diversity and GCS score (Figures 5A,B). On the other hand, a negative correlation was identified between CDR3 aa diversity and hematoma volume (Figures 5C,D). These results collectively indicated that TCR diversity is a potential marker for assessing ICH prognosis.

## Discussion

The CNS parenchyma is considered a lymphocyte-free organ, but T cells are found in the meninges and have been speculated to influence brain function. Emerging evidence insinuates that T cells extravasate from blood vessels into CNS through a chemokine gradient after ICH, similar to the mechanism by which they infiltrate peripheral tissues. Nonetheless, the precise role of T cells in brain function and recovery remains unknown. Earlier studies evinced that the TCR immune repertoire plays a pivotal role in monitoring the immune microenvironment. Accordingly, the identification of the TCR immune repertoire offers a strategy to track alterations in the peripheral immune microenvironment in ICH.

Herein, the immune response in ICH-associated blood was evaluated. The current study identified a significant correlation between the lymphocyte count and symptoms after the onset of ICH, suggesting that ICH can drive a shift in the distribution of peripheral T cells, consistent with the results of prior investigations (9, 18). Indeed, severe brain injury modulates the immune response. However, the precise mechanism by which neurogenic pathways are involved remains undefined. Lymphopenia may be ascribed to spleen shrinkage owing to the synergy between the sympathetic and hypothalamus-pituitary–adrenal axis (18).

A huge pool of evidence indicates that lymphocytes infiltrate into the brain immediately after a stroke (5). Nonetheless, to date, no specific cell population has been identified as a dominant pathogenetic initiator of stroke. While accumulating evidence supports the notion that T cells play a pathological role in brain injuries, the interactions between T cells and brain-intrinsic cells in the brain are poorly understood. Our results revealed the peripheral landscape of TCR change after ICH. Due to TCR tracking the origin of T cell development, the effect of ICH on adaptive immunity could be monitored by TCR repertoire. Moreover, we will find the more clues of the specific T cells in brain injury or repair. The deep mechanism that how the T cells modulate CNS pathological process can be investigated in our further work. A challenge in the identification of a specific T cell subset is determining the factor that triggers T cell diapedesis across the BBB into the lesion site. Recently, regulatory T cells, identified as an immunosuppressive T cell population, have been described as a central cerebroprotective modulator after stroke, targeting multiple inflammatory pathways via the IL-10 signaling pathway (19). Thus, it is of paramount importance to investigate T-cell transformation for the development of an effective immunotherapeutic strategy for ICH patients.

A recent study documented an antigen-independent mechanism of T cell neuroprotection after brain injury. A T cell adoptive treatment in major histocompatibility complex class II (MHC II)-deficient mice markedly alleviated neuronal damage after injury via MYD 88 and IL-4-mediated neuroprotection (20). Although the beneficial effect was independent of TCR-MHC II interaction, T cells may infiltrate the brain after CNS injury via multiple mechanisms (both antigen-dependent and antigen-independent). The availability of antigens or peptides released from the site of brain lesions has been theorized to endow T cells with neuroprotective functions (21). Therefore, tracking TCR repertoire after ICH may offer valuable insights into identifying the origin and function of T cells.

Our study revealed an intact TCR profile of V, J, VJ, VDJ, and CDR3 aa distribution in peripheral blood after ICH. Besides, a centralized clonotype and length CDR3 with chaotic CDR3 motif aa were found in the TCR repertoire, in line with the findings of previous studies reporting that ICH induced a significant decrease in the number of peripheral T cells. The shift in the TCR repertoire is indispensable for antigen recognition after ICH. In addition, notable relationships were discovered between CDR3 diversity and GCS score or hematoma volume, indicating that the TCR repertoire may serve as a biomarker for ICH symptoms and prognosis. Undoubtedly, the ICH-responsive TCR repertoire provides valuable clues on the origin of T cells, which enhances our understanding of the functions of T cells in brain injury. However, the mechanisms that govern TCR reconstitution in CNS damage and repair remain to be unraveled, warranting further studies.

## Data availability statement

The data presented in the study are deposited in the NGDC GSA repository, accession number HRA006678, https://ngdc.cncb.ac.cn/gsa-human/.

## Ethics statement

The studies involving humans were approved by Institutional Ethical Commission for the School of Medicine at Xiamen University. The studies were conducted in accordance with the local legislation and institutional requirements. The participants provided their written informed consent to participate in this study. Written informed consent was obtained from the individual(s) for the publication of any potentially identifiable images or data included in this article.

## Author contributions

RZ: Writing – original draft, Conceptualization, Data curation, Formal analysis, Funding acquisition, Investigation, Methodology, Project administration, Resources, Software, Supervision, Validation, Visualization. LW: Conceptualization, Data curation, Formal analysis, Funding acquisition, Investigation, Methodology, Project administration, Resources, Software, Supervision, Validation, Visualization, Writing – original draft. JZ: Resources, Validation, Visualization, Writing – original draft. XZ: Resources, Validation, Writing – original draft, Formal analysis. PW: Writing – original draft, Writing – review & editing.
