# Supplementary figures and images for: Peripheral T cell immune repertoire is associated with the outcomes of acute spontaneous intracerebral hemorrhage
Source: Front Neurol. 2024 Mar 14;15:1371830. doi: 10.3389/fneur.2024.1371830 (PMC10976974; doi:10.3389/fneur.2024.1371830)

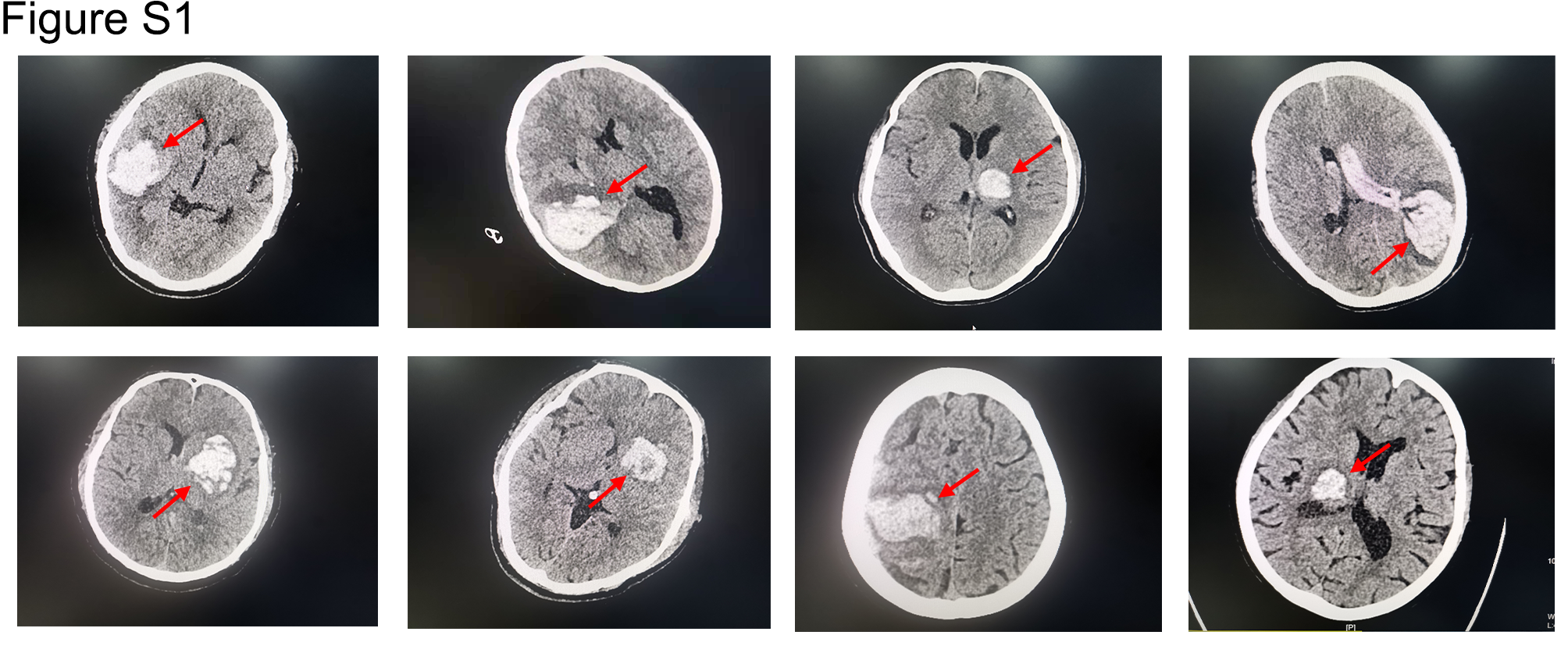

Supplement: Supplementary file 1 [file Image_1.TIF]

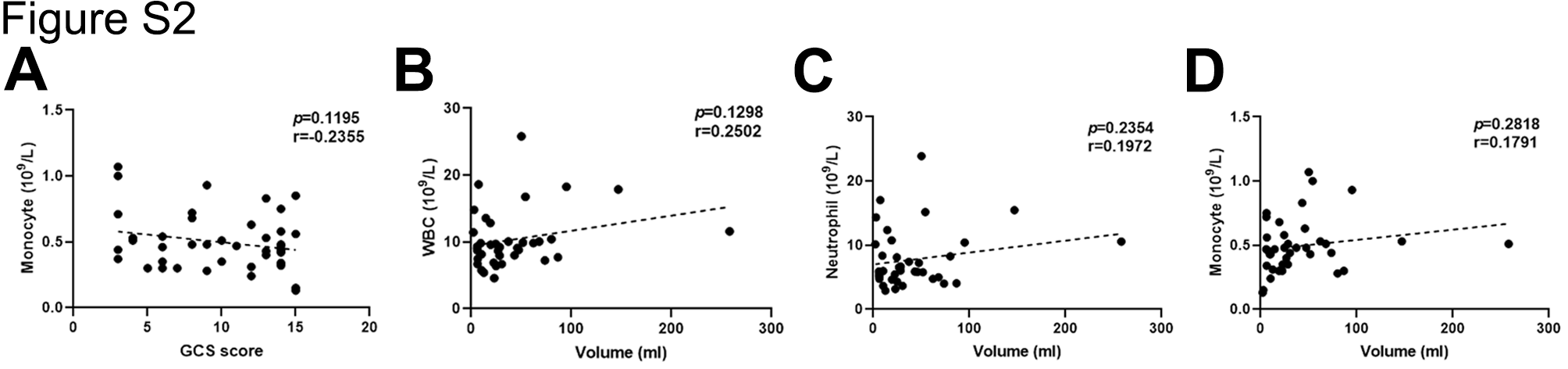

Supplement: Supplementary file 2 [file Image_2.TIF]

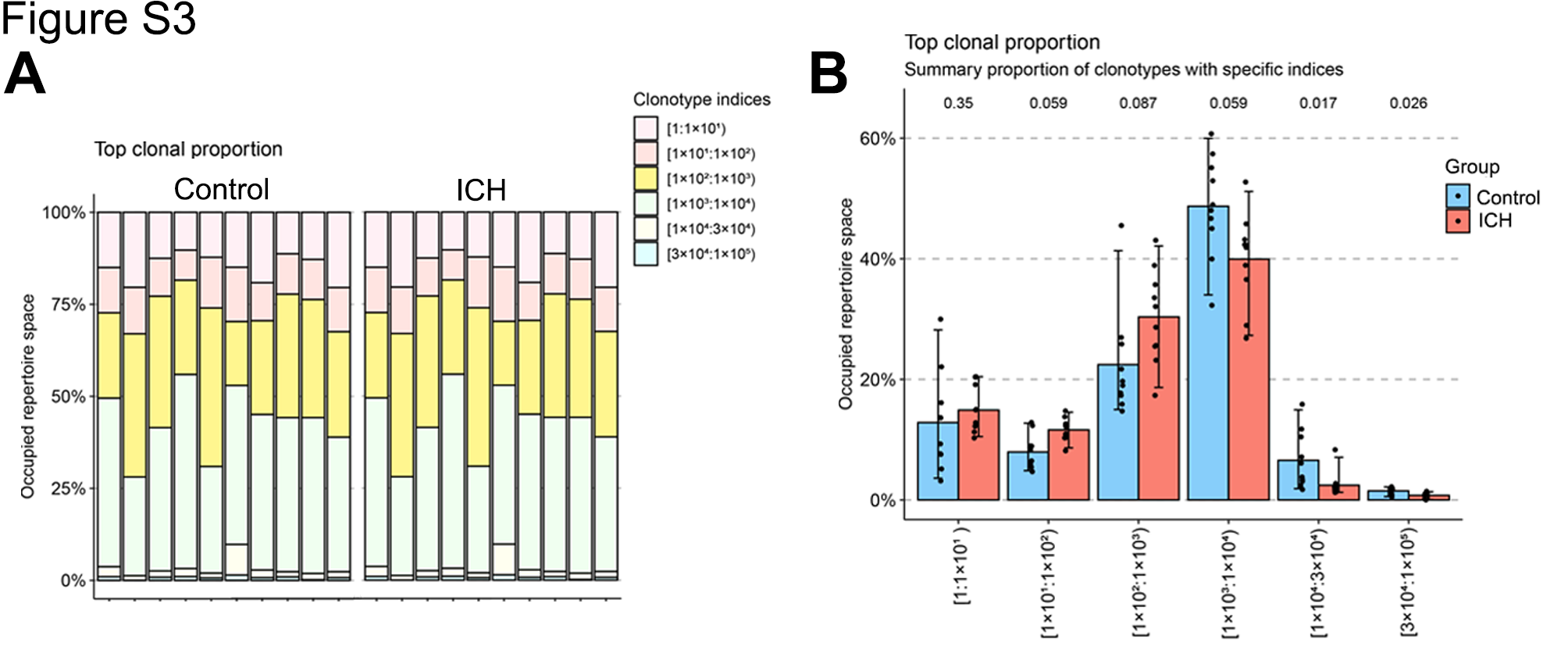

Supplement: Supplementary file 3 [file Image_3.TIF]
